# Different Forms of Selenoprotein M Differentially Affect Aβ Aggregation and ROS Generation

**DOI:** 10.3390/ijms14034385

**Published:** 2013-02-25

**Authors:** Ping Chen, Ruo-Ran Wang, Xiao-Jie Ma, Qiong Liu, Jia-Zuan Ni

**Affiliations:** 1 Shenzhen Key Laboratory of Marine Biotechnology and Ecology, Department of Marine Biology, Shenzhen University, Shenzhen 518060, China; E-Mails: wyf10071121@yahoo.com.cn (P.C.); happymaxiaojie@163.com (X.-J.M.); 2 College of Optoelectronic Engineering, Shenzhen University, Shenzhen 518060, China; 3 Shenzhen Key Laboratory of Microbial Genetic Engineering, College of Life Sciences, Shenzhen University, Shenzhen 518060, China; E-Mail: wangruoran@hotmail.com

**Keywords:** selenoprotein M (SelM), antioxidant, neuroprotection, reactive oxygen species (ROS)

## Abstract

Selenoprotein M (SelM), one of the executants of selenium *in vivo*, is highly expressed in human brain and most probably involved in antioxidation, neuroprotection, and intracellular calcium regulation, which are the key factors for preventing the onset and progression of Alzheimer’s disease (AD). In this paper, human SelM was successfully overexpressed in human embryonic kidney cells HEK293T. Sodium selenite (Na_2_SeO_3_ 0.5 μmol/L) increased the expression of full-length SelM and inhibited the expression of truncated SelM. The full-length SelM exhibited higher antioxidant activity than its selenocysteine-to-cysteine mutation form SelM’, whereas the truncated SelM had an adverse effect that increased the oxidative stress level of cells. When β-amyloid (Aβ_42_, an AD relevant peptide) was cotransfected with the empty expression vector, *SelM*, or *SelM’* under the induction of 0.5 μmol/L Na_2_SeO_3_, the intracellular Aβ_42_ aggregation rates were detected to be 57.9% ± 5.5%, or 22.3% ± 2.6%, or 26.3% ± 2.1%, respectively, showing the inhibitory effects on Aβ aggregation by the full-length SelM and SelM’. Meanwhile, the intumescentia of mitochondria caused by *Aβ_42_* transfection was significantly mitigated by the cotransfection of *SelM* or *SelM’* with *Aβ_42_* under the induction of 0.5 μmol/L Na_2_SeO_3_. On the contrary, cotransfection of *SelM* and *Aβ_42_* without the induction of Na_2_SeO_3_ increased Aβ_42_ aggregation rate to 65.1% ± 3.2%, and it could not inhibit the Aβ-induced intumescent mitochondria. In conclusion, full-length SelM and SelM’ might prevent Aβ aggregation by resisting oxidative stress generated during the formation of Aβ oligomers in cells.

## 1. Introduction

An excessive amount of reactive oxygen species (ROS) is a major cause of cellular damage and death, which has been implicated as a key factor in the early stage of cancer [1], cardiovascular disease [2], and neurodegenerative diseases including Alzheimer’s disease (AD) [3]. Selenium (Se), an essential trace element for mammals, can assist cells to resist oxidative damage. Se *in vivo* is primarily present as various selenoproteins to maintain the balance of the cellular redox state. So far, 25 selenoproteins have been found in humans and 24 in rodents [4]. Most of them play important roles in redox regulation, detoxification, immune-system protection and viral suppression [5]. However, the biological functions of some newly identified selenoproteins remain unknown. Recently, high interest has been focused on the role of Se and selenoproteins in neurodegenerative diseases such as AD [6]. Selenoprotein M (SelM) was first reported as a new selenoprotein via bioinformatics method [4,7]. It is expressed in many human tissues with the highest levels in the brain [7], and may be involved in the onset and development of AD.

Structural studies revealed that SelM had a common redox motif CXXU, where C represents cysteine (Cys) and U represents selenocystein (Sec) [8]. This motif is the redox center of a subset of selenoproteins, for example, selenoprotein H, selenoprotein W, and selenoprotein T [9]. These proteins may function as thiol-disulfide oxidoreductases that participate in the formation of disulfide bonds. They may also function as redox regulators. Overexpression of human SelM in the CMV/GFP-hSelM rat increased the activity of antioxidant enzymes such as glutathione peroxidase (GPx) and superoxide dismutase (SOD), induced a composition change in immune-related cells in response to the addition of 2,2′-azobiz (2-amidinopropane) dihydrochloride (AAPH) [10], and caused a redox shift toward a reduced status in the brain. Reports indicated that SelM expression decreased significantly in neuron-specific tRNA[Ser]^Sec^ (Trsp)-knockout mice brains, leading to neurodegeneration [11]. The down-regulation of the SelM level was correlated with a mouse model of AD that overexpressed a mutated form of human presenilin-2 [12]. Another report also showed that induction of the extracellularly regulated protein kinases (ERK) signaling pathway, which leads to the down-regulation of β/γ secretase-mediated proteolysis or the up-regulation of α secretase-mediated proteolysis and the phosphorylation of Tau protein at several residues, attributed to the overexpression of SelM in rats [13]. SelM has also been reported to have a neuroprotective function through the regulation of cytosolic calcium, which is very important in the pathogenesis of neurodegenerative diseases [14]. All these results indicate that SelM is closely associated with the onset and progression of neurodegenerative diseases, especially AD. Due to the importance of SelM in the brain, the expression of SelM and the study of its mechanism in AD remain the focus and challenge in this field of research. In this paper, different forms of human SelM were expressed in HEK293T cells and their roles on the generation of intracellular ROS and the aggregation of β-amyloid peptide (Aβ_42_, an AD relevant peptide) were compared.

## 2. Results and Discussion

### 2.1. Effect of Sodium Selenite on the Expression Form of SelM

Selenoproteins are a special group of proteins that are notoriously difficult to express by the techniques of gene manipulation due to the special amino acid Sec encoded by a traditional stop codon UGA. The low efficiency of selenoprotein expression holds back their structural and functional study. Decoding UGA to Sec requires a stem-loop structure designated as the Sec insertion sequence (SECIS) element in the 3′-untranslated regions (UTRs) of eukaryotic RNAs. Meanwhile, some trans-acting factors are essential for recoding UGA from a stop signal to a Sec residue [15]. The selenium content of the culture medium is also an important factor for selenoprotein expression. Several methods have been carried out to prepare selenoproteins [16–18], however, most of them are not very efficient.

**Figure 1:**
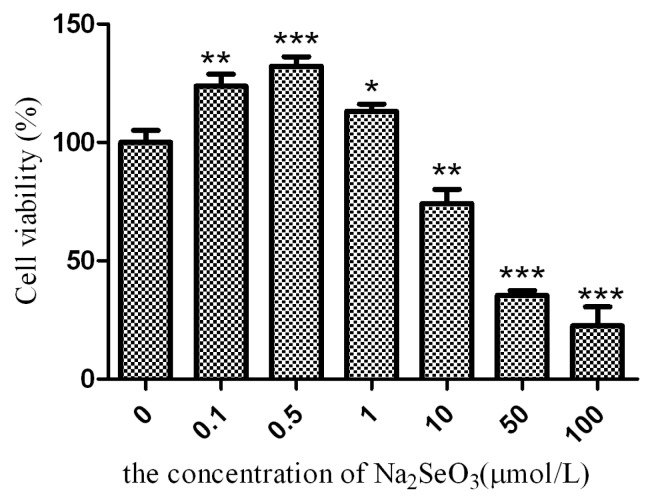
Dose-dependent effect of sodium selenite on the viability of HEK293T cells after 24 h treatment. **p < 0.01 and ***p < 0.001, respectively, indicate significant and very significant differences between the selenite treated and the control cells.

The vector pSelExpress1, which has been reported to overexpress selenoproteins, was constructed containing a SECIS element similar to that in the SelM gene [19]. In this paper, SelM was successfully expressed in HEK293T cells by the pSelExpress1 vector, and its roles in AD were investigated. Previous studies have shown that selenite is double-edged, promoting selenoprotein expression for antioxidation at a low concentration [20,21] and generating oxidative stress at a high level. To find the appropriate concentrations of Na_2_SeO_3_ for either promoting the SelM expression or generating oxidative stress, HEK293T cells were treated with a series of concentrations of sodium selenite for 24 h before viability was measured by the CCK-8 assay. As shown in Figure 1, the viability of HEK293T cells was increased by lower concentrations of Na_2_SeO_3_, but decreased by higher concentrations of Na_2_SeO_3_ (≥10 μmol/L). The optimum concentration to significantly increase the proliferation of HEK293T cells was 0.5 μmol/L, thus this dosage was used to stimulate the expression of SelM in this paper. And 10 μmol/L Na_2_SeO_3_ was used to generate oxidative stress, as this concentration has already shown an inhibitory effect on the cell growth in Figure 1.

**Figure 2:**
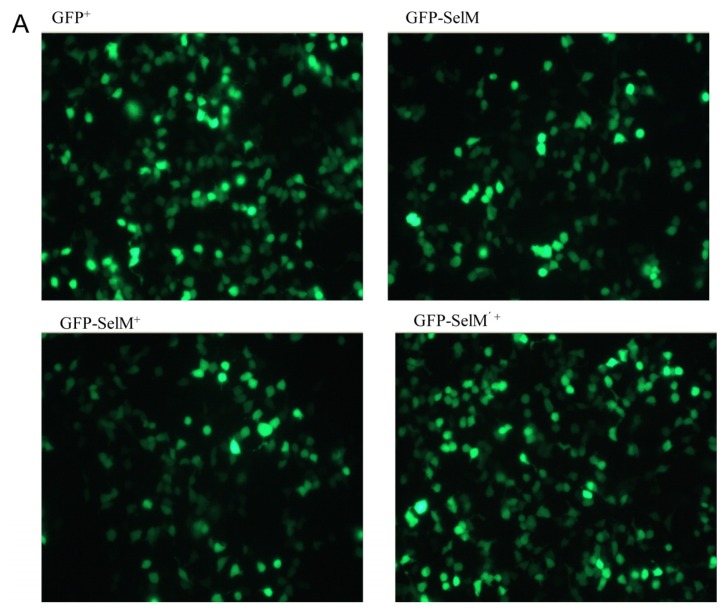

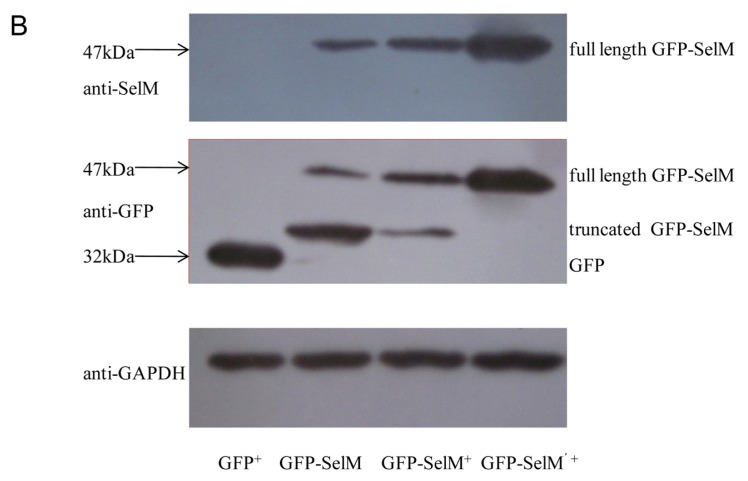
Expression of different forms of human selenoprotein M (SelM) in HERK293T cells. (**A**) Representative GFP fluorescent images (400×). GFP^+^, GFP-SelM^+^, and GFP-SelM’^+^, respectively, indicated those cells transfected with the plasmids of pSelExpress1-*GFP*, pSelExpress1-*GFP-SelM*, and pSelExpress1-*GFP-SelM’* under 0.5 μmol/L Na_2_SeO_3_ treatment. GFP-SelM indicated those cells transfected with the pSelExpress1-*GFP-SelM* plasmid in the absence of Na_2_SeO_3_; (**B**) Detection of different forms of GFP-fused SelM by Western blot analysis using anti-SelM (upper panel), anti-GFP (middle panel), and anti-GAPDH (bottom panel) antibodies.

HEK293T cells transfected with pSelExpress1-*GFP*, pSelExpress1-*GFP-SelM*, or pSelExpress1-*GFP-SelM’* (where the Sec-encoding TGA in *SelM* was site-directedly mutated to Cys-encoding TGC) were treated with or without sodium selenite at a final concentration of 0.5 μmol/L and incubated for 30 h before by fluorescent microscope (Figure 2A) and Western blot (Figure 2B). Figure 2A shows green fluorescence images of those cells transfected with the gene of *GFP* or its fusion proteins. All proteins including GFP, GFP-SelM, and GFP-SelM’ were expressed in HEK293T cells, independent of the fact whether 0.5 μmol/L Na_2_SeO_3_ was added to the culture medium or not (GFP-SelM^+^ in Figure 2A indicates the presence of Na_2_SeO_3_, while GFP-SelM indicates the absence of Na_2_SeO_3_). There is no significant difference in the fluorescence between the GFP-SelM’^+^ and GFP-SelM’ groups (data not shown). As shown in Figure 2B, in the absence of Na_2_SeO_3_, SelM was expressed mainly in the truncated form (the band around 32 kDa in the sodium dodecyl sulfate polyacrylamide gel electrophoresis (SDS-PAGE) gel), while addition of 0.5 μmol/L Na_2_SeO_3_ (*i.e.*, the GFP-SelM^+^ group) increased the expression level of full-length SelM (the band around 47 kDa in the gel) and decreased the expression level of truncated SelM. Transfection of *GFP-SelM’* into the cells only increased the expression of full-length protein, and truncated SelM. was not observed (Figure 2B, the GFP-SelM’^+^ group), demonstrating that the site-directed mutation of TGA to TGC in SelM’ enabled the complete translation of the protein.

Expression of SelM or SelM’ was further verified by Western blot analysis with anti-SelM antibody, which can only recognize the full-length SelM or SelM’ (both bands around 47 kDa) but not the truncated SelM (shown in the upper panel of Figure 2B). Similar to the anti-GFP results, the expression level of full-length SelM detected by anti-SelM antibody increased with the addition of 0.5 μmol/L Na_2_SeO_3_. Meanwhile, the expression level of SelM’ was observed to be much higher than that of SelM, indicating the easy read-through of SelM’ translation with TGA mutated to TGC. Consequently, it can be concluded that 0.5 μmol/L Na_2_SeO_3_ can increase the expression level of full-length SelM while decreasing the level of truncated SelM.

It is well known that the incorporation of Sec into selenoproteins at a UGA codon requires a special tRNA, the selenocysteil tRNA (Sec-tRNA[Ser]^Sec^). When appropriate doses of Na_2_SeO_3_ are provided, the ratio of methylated tRNA[Ser]^Sec^ to its non-methylated form increased. As methylated Sec-tRNASec is the translationally active isoform for the biosynthesis of all selenoproteins, increasing the methylated form with Na_2_SeO_3_ plays an important role in selenoprotein synthesis [22]. Generally, the efficiency of Sec insertion into recombinant proteins is low, and the major products of selenoprotein translation are often the truncated forms. Especially under the condition of limited selenium supply, the UGA codon prefers to be a stop codon, and the truncated form of selenoproteins is easily formed. In this paper, full-length SelM was found to be highly expressed with the addition of 0.5 μmol/L Na_2_SeO_3_, but the truncated SelM became the major form in the absence of Na_2_SeO_3_. Appropriate doses of Na_2_SeO_3_ can promote the expression of full-length SelM and decrease truncated SelM. This result is consistent with previous reports that selenium supplementation is a key factor for the expression of full-length selenoproteins [21].

### 2.2. Effect of Different Forms of SelM on Intracellular ROS and Redox-Regulating Proteins

**Figure 3:**
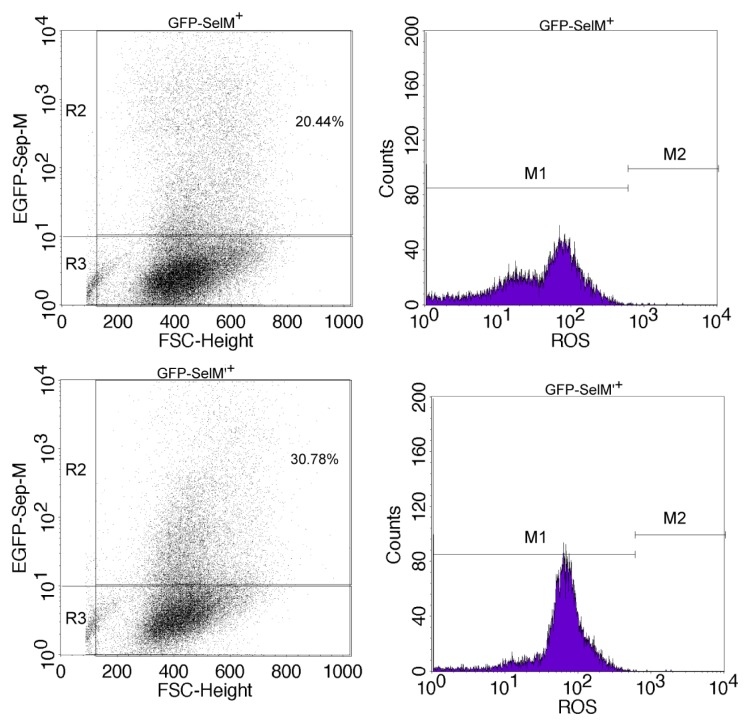

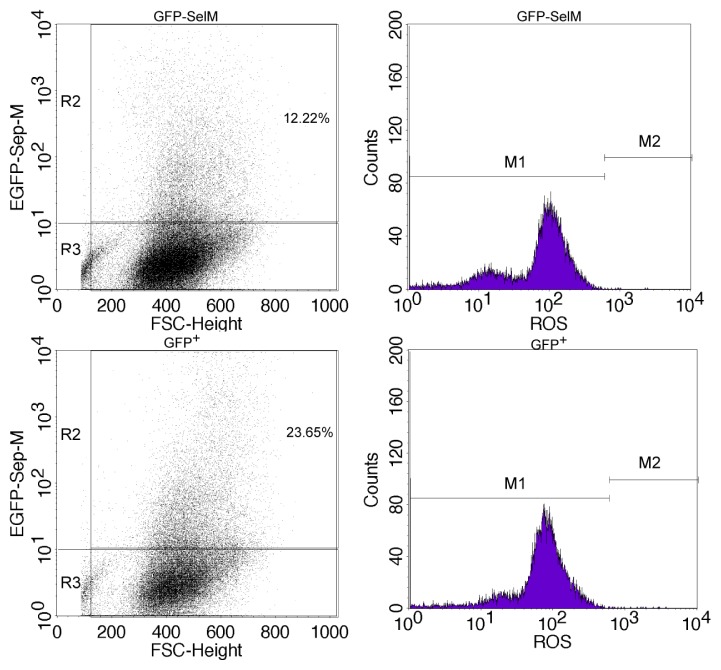
Effect of different forms of SelM on intracellular reactive oxygen species (ROS) generation. HEK293T cells were transfected with different plasmids with or without the addition of 0.5 μmol/L Na_2_SeO_3_, followed by the treatment with 10 μM Na_2_SeO_3_ for 6 h to generate oxidative stress. The transfection efficiency (left panel in each group) and the ROS level (right panel in each group) were measured by flow cytometry. The percentage of transfected cells (R2) *vs.* untransfected cells (R3) is shown in the left panel. GFP-SelM^+^, GFP-SelM’^+^, GFP-SelM, and GFP^+^ represent those cells transfected, respectively the plasmids of pSelExpress1-*GFP-SelM*, pSelExpress1-*GFP-SelM’*, pSelExpress1-*GFP-SelM*, and pSelExpress1-*GFP*. All cells, except those in the GFP-SelM group, were cultured under 0.5 μmol/L Na_2_SeO_3_ for 24 h. This is the one representative profile of three replicates.

**Figure 4:**
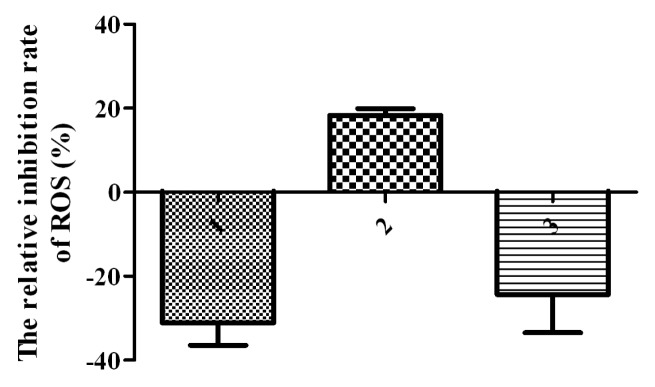
The relative inhibition rate of ROS in the cells transfected with pSelExpress1-*GFP-SelM* under 0.5 μmol/L Na_2_SeO_3_ treatment. (**1**) pSelExpress1-*GFP-SelM*; (**2**) pSelExpress1-*GFP-SelM’* under 0.5 μmol/L Na_2_SeO_3_; (**3**) Cells transfected with pSelExpress1-*GFP* under 0.5 μmol/L Na_2_SeO_3_ were used as a control.

The impact of different forms of SelM on the redox status of HEK293T cells was investigated by the detection of intracellular reactive oxyten species (ROS) levels and redox-regulating protein expression. Cell transfection efficiency was checked through GFP fluorescence with a flow cytometer (Figure 3, right panel in each group). All transfected cells were cultured for 24 h with or without the addition of 0.5 μmol/L Na_2_SeO_3_, followed by the treatment with 10 μmol/L Na_2_SeO_3_ for 6 h and triple phosphate buffer saline (PBS)-wash. Intracellular ROS were detected by applying a probe of dihydroethidium (DHE), an indicator for ROS, into the culture medium and quantified via flow cytometry. As shown in Figure 3, cells transfected with *GFP-SelM* and cultured under 0.5 μmol/L Na_2_SeO_3_ were found to have a significant decrease in ROS levels (indicated as GFP-SelM^+^) compared with the control group (*GFP*-transfected, indicated as GFP^+^), while those cells transfected with *GFP-SelM* without 0.5 μmol/L Na_2_SeO_3_ addition (GFP-SelM) were detected to have a remarkable ROS increase compared with the control. The level of ROS in the *GFP-SelM’*-transfected cells (GFP-SelM’^+^) was in between the two levels of GFP-SelM^+^ and GFP-SelM, and it did not change significantly with or without the addition of 0.5 μmol/L Na_2_SeO_3_. The relative inhibition rates of ROS (RI) executed by different forms of SelM were measured and calculated. Significant ROS inhibition rates were shown in the groups of GFP-SelM and GFP-SelM’ (Figure 4), to both of which 0.5 μmol/L Na_2_SeO_3_ was added. The former group had a higher inhibition rate than the latter. On the other hand, ROS increased markedly in the GFP-SelM-transfected cells in the absence of 0.5 μmol/L Na_2_SeO_3_.

The difference in ROS levels between the GFP-SelM^+^ and GFP-SelM’^+^ groups is mainly due to the mutation of Sec to Cys. SelM and SelM’, respectively, contain a Sec and a Cys residue in the same position. Sec is the Cys-analogue with a sulfur atom replaced by a selenium atom. The major difference between the two amino acids is that Sec is more readily oxidized than Cys, which has a stronger potential to prevent oxidative damage caused by ROS and to reduce tyrosyl radicals in proteins. The lower level of ROS in the GFP-SelM^+^ group compared with the GFP-SelM group is mainly due to the structure change of the protein. SelM has a structural similarity to Trx, an intracellular reducing agent that can directly interact with ROS [23]. The ROS-scavenging effect of the Trx system is realized by the reversible redox motif of CXXC in Trx [24]. Compared with the full-length SelM or its mutant, the truncated SelM has a -Cys-Gly-Gly-COOH motif instead of the thioredoxin-like folding. Only one Cys is present in the truncated SelM, where the Cys residue cannot form a reversible disulfide bond. This Cys can be oxidized easily under aerobic condition, leading to the increase of oxidative stress in cells.

**Figure 5:**
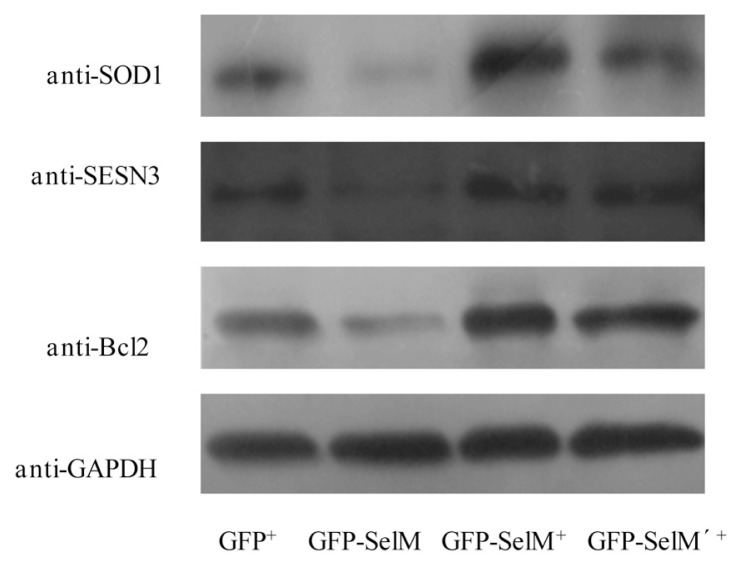
Western blot analysis of the protein level of superoxide dismutase 1 (SOD1), SESN3 and Bcl2. HEK293T cells were transfected with different plasmids with or without the addition of 0.5 μmol/L Na_2_SeO_3_, followed by the treatment with 10 μM Na_2_SeO_3_ for 6 h to generate oxidative stress, GFP^+^, GFP-SelM, GFP-SelM^+^ and GFP-SelM’^+^ represent the same cells as those represented in Figure 3.

As SelM generally has the function of regulating the intracellular redox balance, some other proteins related to oxidative stress and cell growth were also detected. Figure 5 showed that the expression levels of the oxidative stress relevant proteins sestrin 3 (SESN3) SOD1, and cell growth relevant protein Bcl2 increased with the expression of full-length SelM or its mutant SelM’. Transfection of *SelM* or *SelM’* under 0.5 μmol/L Na_2_SeO_3_ could not only increase the expression of SelM or SelM’, but also stimulate other redox-regulating proteins, like SESN3 and SOD1, which functioned together to inhibit intracellular ROS generation. On the other hand, when *SelM* was transfected into cells in the absence of Na_2_SeO_3_, SelM was expressed mainly in the truncated form, where the intracellular ROS level was increased, together with the decreased expression level of redox-regulating proteins. Those results correspond to a previous report that states that the truncated thioredoxin reducatase 1 (TR1) expressed under selenium-deficient condition might rapidly induce cell death, while the enzymatically active full-length TR1 does not [25]. In addition, the truncated SelM lacks the highly flexible *C*-terminal extension (residues 121–145), which plays an important role in undertaking a defined conformation after the binding protein substrates or other redox proteins [8]. These may be the reasons why full-length SelM has an antioxidative function, while the truncated SelM has an adverse effect.

### 2.3. Effect of Different Forms of SelM on Aβ Aggregation

**Figure 6:**
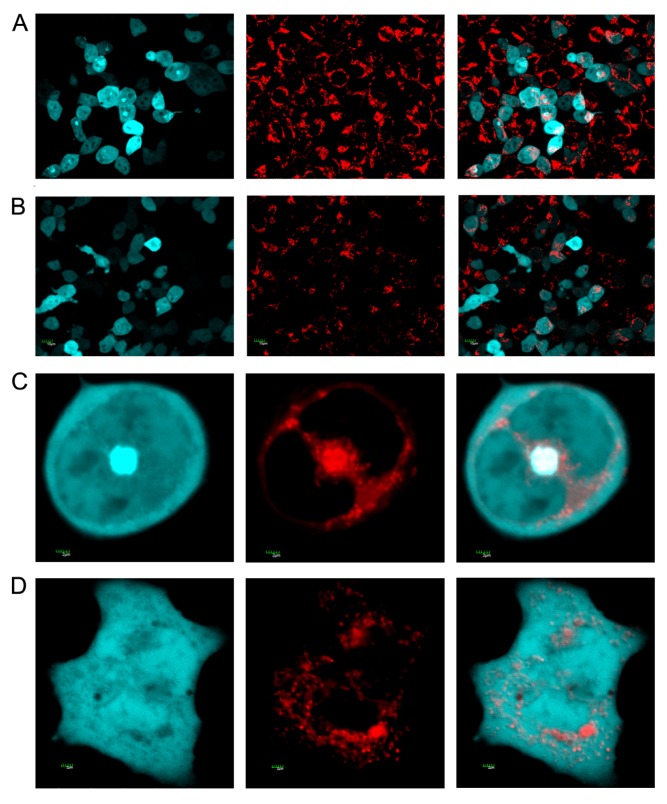
The mitochondrial morphology and location of Aβ_42_-CFP fusion proteins inside cells. The figures shown here are representative ones. HEK293T cells were cotranfected with *Aβ_42_-CFP* plasmid and pSelexpress vacant vector (**A** & **C**) or pSelexpress-*SelM*-myc (**B** & **D**), under 0.5 μmol/L Na_2_SeO_3_ treatment. Left panels show cyan fluorescence from the CFP-fusion proteins; center panels are mitochondria images stained with Mito-Tracker Red CMXRos; right panels are images merging the left and the center. Scale bar in **A** & **B**: 10 μm and **C** & **D**: 2 μm.

**Figure 7:**
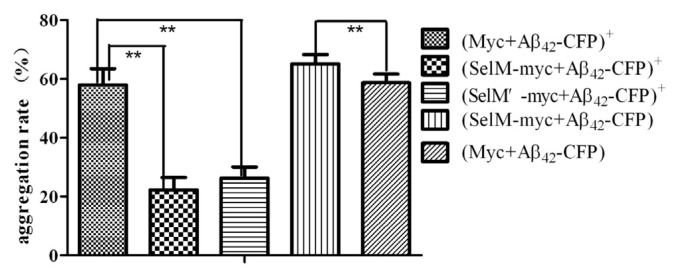
The aggregation rate of Aβ_42_-CFP. HEK293T cells were cotransfected with *Aβ_42_-CFP* and *SelM-myc* or *SelM’-myc*. The data represent the mean ± SD from three replicates. **p < 0.01 indicates significant differences compared to the cells cotransfected with the pSeleExpress1 vacant vector and *Aβ_42_-CFP* plasmid.

SelM has been reported to be closely associated with AD [11–13]. To further investigate the impact of different forms of SelM on the aggregation of Aβ_42_, plasmids containing *Aβ_42_-CFP* and *SelM-myc* (or *SelM’-myc*) gene fragments were transiently cotransfected into HEK293T cells, cultured for 36 h, and analyzed by laser confocal microscopy (Figure 6A). Meanwhile, the *Aβ_42_-CFP* plasmid and pSeleExpress1 vacant vector were cotransfected into the cells as a control (Figure 6B). Aβ_42_ has been known to be extremely insoluble *in vitro*, and the intracellular Aβ forms perinuclear aggregates in the cytosol of HEK293T cells [26]. In the present study, fusion protein Aβ_42_-CFP in most cells cotransfected with *Aβ_42_-CFP* plasmid and pSeleExpress1 vacant vector ((Myc+Aβ_42_-CFP)^+^ group in Figure 7) aggregated largely in the cytosol (Figure 6A). By counting around 300 cells including Aβ_42_ aggregated and non-aggregated in three independent experiments, the aggregation rate (*i.e.*, the ratio of aggregated cell number to the total cell number) [27] was calculated to be 57.9% ± 5.5% in the (Myc + Aβ_42_-CFP)^+^ group (Figure 7). However, fusion protein Aβ_42_-CFP in the cells cotransfected with *Aβ_42_-CFP* and *SelM-myc* plasmids under 0.5 μmol/L Na_2_SeO_3_ (the (SelM + Aβ_42_-CFP)^+^ group) were evenly distributed in most cells, and the aggregation rate was calculated as only 22.3% ± 2.6%. Similar images of Aβ_42_-CFP were also observed in the cells cotransfected with *Aβ_42_-CFP* and *SelM’-myc* plasmids (the (SelM’ + Aβ_42_-CFP)^+^ group) with the aggregation rate of 26.3% ± 3.8%. Contrarily, Aβ_42_-CFP in the cells cotransfected with *Aβ_42_-CFP* and *SelM-myc* without the addition of 0.5 μmol/L Na_2_SeO_3_ (the (SelM + Aβ_42_-CFP) group) aggregated largely with the aggregation rate of 65.1% ± 3.2%, which was even higher than that of the control cells. Since SelM-transfected cells mainly expressed full-length SelM in the presence of 0.5 μmol/L Na_2_SeO_3_ and the truncated SelM in the absence of Na_2_SeO_3_, it can be concluded that full-length SelM, together with its mutant SelM’, play important roles in preventing the aggregation of Aβ_42_, while the truncated SelM has an adverse effect, which slightly promotes the aggregation of Aβ_42_.

It is well known that mitochondria play important roles in the pathogenesis of neurodegenerative diseases [26,28,29]. In normal cells, mitochondria are primarily distributed at the cytoplasmatic edge and are always in the filamentous state. However, the mitochondria of *Aβ_42_-CFP*-transfected cells are mainly concentrated in the areas where Aβ gathers, and their morphology appears as an intumescent phenomenon (Figure 6C). However, the morphology of mitochondria is normal in most cells when full-length *SelM* or *SelM’* are co-transfected with *Aβ_42_-CFP* (Figure 6D).

Aβ related mitochondrial dysfunctions, including the mutation of mitochondrial DNA, suppression of mitochondrial respiratory chain activity, decrease of glucose metabolism, deactivation of some key enzymes, increase of ROS generation, and perturbation of calcium homeostasis, have been observed in AD patients, AD animal models, or Aβ-treated cell cultures [30]. In our study, when *Aβ_42_-CFP* and pSeleExpress1 vacant vector were cotransfected into the cells, most of the expressed Aβ_42_ presented in the aggregate state, as did the mitochondria. But when *Aβ_42_-CFP* was cotransfected with *SelM* or *SelM’* under the treatment of a low dosage of sodium selenite, Aβ aggregation was evidently decreased. Meanwhile the distribution and morphology of mitochondria also tended to be normal. This phenomenon did not occur in the cells cotransfected with *Aβ_42_-CFP* and SelM without selenite addition. In the absence of selenite, Aβ aggregation increased and the distribution form of mitochondria also turned out to be abnormal. The impairment of mitochochondrial morphology and location induced by Aβ_42_ were largely prevented in the cells cotransfected with SelM or SelM’, indicating that SelM or its mutant plays an important role in restraining the aggregation of Aβ_42_. The phenomena above are mainly due to the oxidative stress-mediating competence of different forms of SelM. Full-length SelM and SelM’ can restrain the increase of intracellular ROS level, while the truncated SelM has an adverse effect.

## 3. Experimental Section

### 3.1. Plasmid Construction

Primers were designed according to the information of *SelM* (NM_080430.2) in the National Center for Biotechnology Information (NCBI) database to amplify the ORF of *SelM* from the human HEK293T cell lines using the reverse transcription polymerase chain reaction (RT-PCR) method. *SelM’*, the Sec-to-Cys mutated SelM, was amplified by the overlapping PCR method [31]. Both *SelM* and *SelM’* fragments were inserted separately into the pMD18-T vector to generate the plasmids of pMD18-T-*SelM* and pMD18-T-*SelM,*. The DNA fragments of GFP and its fused proteins, including *GFP, GFP-SelM*, and *GFP-SelM’*, were generated and inserted into the plasmid pSelExpress1 at the digestion sites of *Sal*I and *Xba*I with their respective pairs of primers. *SelM-myc* and *SelM’-myc* were amplified from the constructs pMD18-T-*SelM* and pMD18-T-*SelM’,* respectively, and inserted into the pSelExpress1 plasmid at the same double-enzyme digestion sites. *Aβ_42_-CFP* was amplified from pCDF-*Aβ_42_-GFP* and pECFP-C1, and then subcloned into the pCDNA3.1(+) vector at the digestion sites of *NheI* and *EcoRV* to produce the construct of pCDNA3.1(+)-*Aβ_42_-CFP*. Primers used and plasmids used in this paper are all presented in Table 1. DNA sequencing was performed (Invitrogen Biotech, Guangzhou, China) to check the correction of each sequence inserted into the plasmid.

### 3.2. Cell Culture and Transfection

Human embryonic kidney (HEK) 293T cells were cultured at 37 °C a humidified 5% CO_2_ incubator in the Dulbecco’s Modified Eagle Minimum Essential Medium (DMEM) (Invitrogen, Guangzhou, China) supplemented with 10% (*v*/*v*) heat-inactivated fetal bovine serum (FBS) (Invitrogen, Guangzhou, China), 100 units/mL penicillin, and 100 units/mL streptomycin (Invitrogen, Guangzhou, China). Cells were seeded in six-well plates or 35-mm Petri dishes. Plasmids were transfected into HEK293T cells using Lipofectamin and Plus reagent kit (Invitrogen, Guangzhou, China) according to the manufacturer’s instruction.

### 3.3. Cell Viability Assay

HEK293T cells (1 × 10^3^ per well) were plated in 96-well microplates. After treatment with a series of final dosages of sodium selenite (0.1, 0.5, 1, 10, 50, and 100 μmol/L in FBS-free DMEM) for 24 h, 10% CCK-8 (Beyotime, Shanghai, China) was added to each well for an additional 2 h. The absorbance of the sample was measured at 450 nm wavelength with a reference wavelength of 650 nm using a SpecTRA MAX 190 microplate reader (Molecular Devices, Sunnyvale, CA, USA) to detect cell viability by CCK-8 assay [32]. Cells without Na_2_SeO_3_ treatment served as the control. Triplicates were performed throughout the procedures.

### 3.4. Staining of Mitochondria

Cells cotransfected with the plasmids of pECFP-*Aβ_42_* and pSelExpress1-*SelM-myc* or pSelExpress1-*SelM’-myc* or pSelExpress1 vacant vector were incubated in the incubator for 24–36 h, washed three times with pre-warmed PBS, incubated in the 0.3 μmol/L MitoTracker (Invitrogen, Guangzhou, China) medium for 15 min, discarded the supernatant, and incubated with pre-warmed PBS (37 °C) for 10 min to remove unbound probe. Pre-warmed 3.7% paraformaldehyde was used to fix the cells for 15 min. Then the cells were washed with PBS three times and photographed by laser confocal microscopy (Olympus FluoView 1000, Olympus corpopration: Tokyo, Japan).

### 3.5. Western Blot Analysis

Western blot analysis was performed using primary antibodies (Abs) against GFP and myc-tag (Abmart, Shannghai, China), Bcl-2, SESN3 and GAPDH (Santa Cruz Biotech, Santa Cruz, CA, USA), SOD1 (Santa Cruz Biotech, Santa Cruz, CA, USA), SelM and Rac1 (Abcam, Cambridge, UK) at optimized dilutions. Total protein extracts were separated by sodium dodecyl sulphate-polyacrylamide gel elctrophoresis (SDS-PAGE) with 10% or 15% acrylamide separating gel. GAPDH was used for the normalization of each protein to ensure equal amount of protein was loaded to the gel and transferred to polyvinylidene fluoride (PVDF) membranes (Millipore, Madison, WI, USA). The blots were incubated overnight at 4 °C with the primary Ab. After three-time wash with tris-buffered saline with Tween (TBS-Tween), those blots were incubated for 1h at 25 °C with the secondary Ab. After a further wash, the immune complexes were revealed by enhanced chemiluminescence (ECL) (Pierce ECL detection kit, Thermo Fisher Scientific Inc., Rockford, IL, USA).

### 3.6. Measurement of Intracellular ROS Level

The levels of intracellular ROS were determined using a ROS assay kit (Vigorous Biotech, Beijing, China), following the manufacturer’s protocol. Briefly, pSelExpress1-*GFP^+^*, pSelExpress1-*GFP-SelM’^+^*, pSelExpress1-*GFP-SelM^+^* and pSelExpress1-*GFP-SelM* transfected cells in six-well plate were incubated in DMEM medium for 24 h, and subsequently treated with 10 μmol/L Na_2_SeO_3_ for 6 h. The cells were then harvested and incubated with 20 μmol/L dihydroethidium (DHE) at room temperature for 30 min in the dark and analyzed using a flow cytometer (Beckman Coulter Altra). The fluorescence intensity was monitored at an excitation wavelength of 488 nm and an emission wavelength of 605 nm, and at the same time, the transfection efficiency of each group was detected by flow cytometry according to the expression level of GFP fused to the proteins. The relative inhibition rates of ROS (RI) were calculated according to the equation, $RI=\frac{I_{n}-I_{0}}{I_{0}}\times 100\%$, where *I_n_* represents the level of ROS in the cells transfected with different forms of fusion SelM (the GFP-SelM, GFP-SelM^+^, and GFP-SelM’ groups), and *I*_0_ represents that of the control (the GFP^+^ group).

### 3.7. Statistical Analysis

Statistical analysis was performed using two-tailed Student’s *t*-tests, *p* < 0.01 and *p* < 0.001 were considered as significant and very significant differences, respectively. Data were expressed as the mean ± SD of triplicate samples. All results were confirmed in at least three independent experiments.

## 4. Conclusions

In conclusion, Na_2_SeO_3_ at proper doses can induce the expression of full-length SelM in HEK293T cells. Full-length SelM or its Sec-to-Cys mutant SelM’ can inhibit intracellular ROS generation, protect mitochondrial, and prevent Aβ aggregation. However, the truncated SelM has an adverse effect. This study provides a mechanism for selenium to prevent AD in its early stage.

## Figures and Tables

**Table 1:** Primers used and plasmids constructed.

Primer	Sequence	Restriction site	Gene fragment	Plasmid constructed
F1	5′-GCCACTGCCTACCGGCCGGAC-3′		SelM ORF cutting off the first 69 bp sequence coding for a signal peptide	pMD18T-*SelM*
R1	5′-CTACAGGTCAGCGTGGTCCGAAG-3′			

F1	5′-GCCACTGCCTACCGGCCGGAC-3′		SelM ORF cutting off the first 69 bp sequence coding for a signal peptide and creating the mutation site from TGA to TGC	pMD18T-*SelM’*
R1	5′-CTACAGGTCAGCGTGGTCCGAAG-3′			
F2	5′-GCGGGGGATGCCAGCTGAAC-3′			
R2	5′-GTTCAGCTGGCATCCCCCGC-3′			

F3	5′-ACGCGTCGACATGGTGAGCAAGGG-3′	*SalI*	GFP ORF (following a stop codon) fused with SelM ORF mentioned above	pSelExpress1-*GFP*
R3	5′-CGGAATTCTTACTTGTACAGCTCGTCCATG-3′	*ECoRI*		
F4	5′-CGGAATTCGCCACTGCCTACCGG-3′	*ECoRI*		
R4	5′-GCTCTAGACTACAGGTCAGCGTGG-3′	*XbaI*		

F3	5′-ACGCGTCGACATGGTGAGCAAGGG-3′	*SalI*	GFP ORF fused with SelM ORF or its mutant mentioned above	pSelExpress1-*GFP-SelM* or pSelExpress1-*GFP-SelM’*
R5	5′-CGGAATTCCTTGTACAGCTCGTCCATG-3′	*ECoRI*		
F4	5′-CGGAATTCGCCACTGCCTACCGG-3′	*ECoRI*		
R4	5′-GCTCTAGACTACAGGTCAGCGTGG-3′	*XbaI*		

F5	5′-ACGCGTCGACGCCACTGCCTACCGG-3′	*SalI*	SelM or its mutant mentioned above	pSelExpress1-*SelM-myc* or pSelExpress1-*SelM’*-myc
R6	5′-GCTCTAGACTACAGATCCTCTTCAGAGAT GAGTTTCTGCTCCAGGTCAGCGTGGTCCG-3′	*XbaI*		

F6	5′-CTAGCTAGCGCCACCATGGATGCGGAAT TTCGCCAT-3′	*NheI*	Aβ_42_ fused CFP	pCDNA3.1(+)-*Aβ_42_*-CFP
R7	5′-GGAATTCCATATGGGATTCGCCAG-3′	*NdeI*		
F7	5′-GGAATTCCATATGGTGAGCAAGGGCGAG-3′	*NdeI*		
R8	5′-CCGGATATCTTACTTGTACAGCTCGT-3′	*EcoRV*		

Underlined sequences are digestion sites of DNA restriction endonuclease.
